# Association between food insecurity and anemia among women of reproductive age

**DOI:** 10.7717/peerj.1945

**Published:** 2016-05-05

**Authors:** Bishwajit Ghose, Shangfeng Tang, Sanni Yaya, Zhanchun Feng

**Affiliations:** 1 School of Medicine and Health Management, Center for Health Research Service in Rural Areas, Key Research Institute of Humanities & Social Sciences at Universities in Hubei Province, Tongji Medical College, Huazhong University of Science and Technology, Wuhan, China; 2 Faculty of Social Sciences, University of Ottawa, Ottawa, Canada

**Keywords:** Women, Anemia, Bangladesh, Micronutrient deficiency, Food insecurity

## Abstract

**Background:** Food insecurity and hidden hunger (micronutrient deficiency) affect about two billion people globally. Household food insecurity (HFI) has been shown to be associated with one or multiple micronutrient (MMN) deficiencies among women and children. Chronic food insecurity leads to various deficiency disorders, among which anemia stands out as the most prevalent one. As a high malnutrition prevalent country, Bangladesh has one of the highest rates of anemia among all Asian countries. In this study, we wanted to investigate for any association exists between HFI and anemia among women of reproductive age in Bangladesh.

**Methodology:** Information about demographics, socioeconomic and anemia status on 5,666 married women ageing between 13 and 40 years were collected from a nationally representative cross-sectional survey Bangladesh Demographic and Health Survey (BDHS 2011). Food security was measured by the Household Food Insecurity Access Scale (HFIAS). Capillary hemoglobin concentration (Hb) measured by HemoCue® was used as the biomarker of anemia. Data were analysed using cross-tabulation, chi-square tests and multiple logistic regression methods.

**Results:** Anemia prevalence was 41.7%. Logistic regression showed statistically significant association with anemia and type of residency (p = 0.459; OR = 0.953, 95%CI = 0.840–1.082), wealth status (Poorest: p < 0.001; OR = 1.369, 95%CI = 1.176–1.594; and average: p = 0.030; 95%CI = 1.017–1.398), educational attainment (p < 0.001; OR = 1.276, 95%CI = 1.132–1.439) and household food insecurity (p < 0.001; 95%CI = 1.348–1.830). Women who reported food insecurity were about 1.6 times more likely to suffer from anemia compared to their food secure counterparts.

**Conclusion:** HFI is a significant predictor of anemia among women of reproductive age in Bangladesh. Programs targeting HFI could prove beneficial for anemia reduction strategies. Gender aspects of food and nutrition insecurity should be taken into consideration in designing national anemia prevention frameworks.

## Introduction

The aim of the Millennium Declaration agenda was to address the most pressing issues regarding human development and consisted of eight broad goals (United Nations Development Program, 2015) which were subdivided into measurable targets and indicators of progress. The first of the eight goals was dedicated to: (1) Reduce by half the proportion of people living on less than $1.25 a day, and (2) Reduce by half the proportion of population who suffers from hunger (United Nations Development Program, 2015). However, achievement towards these goals remain far from becoming a reality as hunger-related causes continue to claim about 25,000 lives a day including one child every 5 s (Sheeran, 2008). The statistics on hidden hunger or micronutrient deficiency reveal a more harrowing scenario since it affects around two billion people globally (Muthayya et al., 2013).

As the home to world’s second largest poor and malnourished population, countries in South Asia suffer extraordinary challenges in population health stemming to a large part from widespread hunger and micronutrient deficiency disorders mainly from vitamins A and D, iron, iodine and zinc (Akhtar, 2015). In Bangladesh, micronutrient deficiency disorders constitute a major public health challenge especially for women and children (Jamil et al., 2008). Literature review on food insecurity and anemia suggests that there has been a growing interest surrounding food insecurity and macronutrient deficiency issues (particularly anemia) in the context of women and infant health (Darnton-Hill & Mkparu, 2015; Torheim et al., 2010). The impact of food insecurity and anemia has been shown to be more pronounced among women of reproductive age especially for pregnant mothers due to their increased requirement of micronutrients (Scholl, 2005). Anemia is also reported to be the most prevalent nutritional deficiency that affect pregnancy outcome and threaten the life of both mother and fetus (Olson, 2010). One American study found significant association between HFI and anemia on adolescents of both sex (Eicher-Miller et al., 2009), but not on pregnant women in North Carolina (Laraia, Siega-Riz & Gundersen, 2010). While in Mexico, HFI was reported to be significantly associated with anemia among adult women, but not among adolescent women (Fischer et al., 2014).

Bangladesh has historically been a high anemia prevalent country with a higher proportion in the rural areas. About three-quarter of the total population were reportedly suffering from varying degrees of anemia during 1975/76, among which more than three-fourth were rural residents; while in 1995/96, the rural prevalence was around 45% among adolescent and non-pregnant women and 50% among pregnant mothers (Ahmed, 2000). The prevalence among rural non-pregnant women slightly reduced to 43.5% in 2010–2011 against an urban rate of 37.2% (Kamruzzaman et al., 2015). Apart from a high burden of anemia, undernutrition also appears to be a widespread phenomenon among Bangladeshi women with about one-third of all women currently living with a body mass index (BMI) below 18.5 kg/m^2^ (Ahmed et al., 2012). Over the past few decades, the situation of undernutrition among women has attracted increased political attention led by the growing understanding of the gender dimensions of food security and the impact of women’s socioeconomic vulnerability on their lack of control over personal nutritional and other health issues (Ivers & Cullen, 2011). Gender differentials in income and food poverty are also known in the country for a long time. In addition to persistent income inequality (Chowdhury et al., 2013), household dietary surveys have demonstrated that male members usually consume more calories and nutritious foods and have a higher frequency of health care services utilization compared to women (Chen, Huq & D’Souza, 1981). Poverty and income inequality exert a direct influence on the two main pillars of food security: accessibility and availability (Varadharajan, Thomas & Kurpad, 2013), and their individual or combined effects are likely to present hard trade-offs between food and other household and personal necessities, and thus set the conditions for food vulnerability and increased susceptibility to micronutrient deficiency diseases. In recent years, the Bangladesh government has made several programmatic efforts to address anemia among women (e.g. iron and folic acid/IFA supplementation) especially in the rural areas. Population based studies focusing on the actors that affect anemia can contribute to generating valuable insights necessary for evidence based health policymaking. In this study, we aimed to explore this association in the context of women of reproductive age in Bangladesh.

## Methodology

### 

#### Study area and data collection

The survey was conducted in all seven administrative regions in Bangladesh. Data were collected from the sixth round (latest) of Bangladesh Demographic and Health Survey (BDHS 2011). The cross-sectional survey was carried out by the National Institute of Population Research and Training (NIPORT) as a part of the International Demographic and Health Survey program known as MEASURE DHS. The program is conducted under the auspices of the United State Agency for International Development (USAID) by the technical assistance of ICF International of Calverton based in USA.

### Variables selection and measurement

#### Dependent variable

Anemia status was the dependent variable in this study. Based on hemoglobin concentration (g/dL), World Health Organization (WHO) guidelines were followed to measure anemia status. Stratification of anemia status was limited to anemic (Hb < 11 g/dl; mild/moderate/severe) and non-anemic (Hb ≥ 11 g/dl) due to low proportion of ‘moderate’ (5.8%) and very low proportion of ‘severe anemia’ (0.2%) against the mild category (35.4%). HemoCue® blood hemoglobin testing system was employed (by trained surveyors) through finger prick method. HemoCue® (HemoCue Inc., Mission Viejo, CA, USA) is a user friendly and highly reliable point-of-care testing (POCT) system and one of the most commonly utilized of hemoglobin testing devices (National Institute of Population Research and Training, 2013).

Food insecurity was the explanatory variable of primary interest. DHS employs the Household Food Insecurity Access Scale (HFIAS) to measure the prevalence and degree of household food insecurity. Originally developed by USAID funded Food and Nutrition Technical Assistance (FANTA) project (National Institute of Population Research and Training, 2013), the scaling system is based on responses to the following yes/no questions: 1) Had three square meals in the past 12 months, 2) Skipped entire meals in the past 12 months, 3) Ate less food in the past 12 months, 4) Ate wheat or rice in the past 12 months, 5) Asked food from relatives or neighbors in the past 12 months. For this study, HFI status was dichotomized into two broad groups in the following manner: ‘Secure’ = Said yes to none of the above-mentioned questions; and ‘Food insecure’ = Said yes to any/all of the above-mentioned questions.

Covariates (Socioeconomic and demographic) were categorized in the following way:

1. Age: < 25 years, and ≥ 25 years; 2. type of residency: rural and urban; 3. educational attainment: < 6 years (nil to below primary level), and ≥ 6 years (primary and above); 4. Wealth status: {Lowest (below average)} (Detailed explanation of measurement of wealth status are available in the report (National Institute of Population Research and Training, 2013)), {Middle (average)}, Highest {(below average}; 5. Employment status (Yes/No); 6. Microcredit borrower (Yes/No) (based on membership with any of the following institutions 1. Association for Social Advancement aka (ASA), 2. Bangladesh Rural Advancement Committee aka (BRAC), 3. Bangladesh Rural Development Board aka (BRDB). 4. Grameen Bank; 7. BMI: Normal weight (18.5–25), Underweight (< 18.5), Overweight (25–30), Obese (> 30).

### Data analysis

Socioeconomic characteristics were presented using descriptive statistics and group comparisons (Anemic or non-anemic) were shown by cross-tabulation. Pearson’s Chi-square tests were performed to check for statistical association between anemic and non-anemic groups for food security status and the socioeconomic variables. All the dependent and independent variables were categorized to facilitate the analysis. Binary logistic regression (generalized estimating equations) method was used to identify the factors of independent association with anemia status. Results of regression analysis were presented as p-values and odds ratios. All tests were two-tailed, and statistical significance was set at p-value less than 0.05. Data analyses were performed with SPSS 20 for Mac (SPSS Inc., Chicago, IL, USA).

### Ethical clearance

The DHS Program surveys maintains strict protocols to maintain ethical standards at all levels of the survey. All participants are required to provide informed consent in order to be eligible for the interview. The consent form is appended below the reference section. In addition, the ICF International ensures that the survey complies with the U.S. Department of Health and Human Services regulations for the protection of human subjects, and the host country ensures that the survey complies with laws and norms of the nation. Further approval for this study was not required since the data is secondary and was made available in the public domain upon registration. More details regarding DHS data and ethical standards are available at: http://goo.gl/ny8T6X.

## Results

### Baseline characteristics

Mean age of the sample population was 31.4 (SD 9.33) years. Table 1 shows that about one-third of the women (33.4) were aged below 25 years and about two-third were of rural origin (65.2%). Women from richest category comprised 44.7% of the sample population, while 36.3% reported living in poor conditions. A quarter of the women had no formal education (25.7%) and about two-third had completed primary level education (74.3%). Only 13.5% women had outdoor employment and more than one-fourth were microcredit borrowers (27.4%). Regarding BMI, 60.2% of the participants were normal weight, (15 deleted) about a one-fourth (24.5%) were underweight and 15.3% were overweight/obese. More than four-fifth (81.6%) of the women reported living in food secure conditions and little less than in food insecure conditions (18.4%).

**Table 1:** Basic characteristics of the sample population (n = 5,666) (Bangladesh Demographic and Household Survey, 2011: National Institute of Population Research and Training, 2013).

Variable	Frequency	Percentage
**Age (31.4 ± 9.33)**		
< 25	1,895	33.4
≥ 25	3,771	66.6
**Residence**		
Urban	1,970	34.8
Rural	3,696	65.2
**Wealth status**		
Poorest	2,056	36.3
Middle	1,078	19.0
Richest	2,532	44.7
**Educational attainment**		
< 6 years	1,458	25.7
≥ 6 years	4,208	74.3
**Employed**		
Yes	767	13.5
No	4,899	86.5
**Microcredit borrower**		
Yes	1,553	27.4
No	4,113	72.6
**BMI category (kg/m^2^)**		
Normal (18.5–25)	3,412	60.2
Underweight (< 18.5)	1,388	24.5
Overweight/Obese (> 25)	866	15.3
**Food security status**		
Secure	4,626	81.6
Insecure	1,040	18.4

### Cross-tabulation and chi-square tests

Table 2 shows the results of cross-tabulation between the two groups based on their anemia status and its association with the explanatory variables. Irrespective of the degree of severity, prevalence of anemia was 41.7% in the sample population. Results show that women who lived in the poorest families, had less than six years of formal education were more likely to be anemic. Participants who had microcredit membership, had BMI above 25, and were food secure had less likelihood of being anemic.

**Table 2:** Prevalence of anemia across the sociodemographic and household variables (Bangladesh Demographic and Household Survey, 2011: National Institute of Population Research and Training, 2013).

Variables	Anemic		p-value
	Yes (41.7%)	No (58.3%)	
**Age**			0.064
< 25	32.3	34.3	
≥ 25	67.7	65.7	
**Residence**			< 0.001*
Urban	31.0	37.4	
Rural	69.0	62.6	
**Wealth status**			< 0.001*
Poorest	41.8	32.3	
Middle	19.7	18.5	
Richest	38.5	49.1	
**Educational attainment**			< 0.001*
< 6 years	76.2	71.5	
≥ 6 years	23.8	28.5	
**Employed**			0.144
Yes	14.1	13.1	
No	85.9	86.9	
**Food security level**			< 0.001*
Secure	13.7	21.7	
Insecure	86.3	78.3	
**Microcredit membership**			0.001*
Yes	29.6	25.9	
No	70.4	74.1	
**BMI category**			0.009*
Normal (18.5–25)	57.9	61.9	
Underweight (< 18.5)	26.2	23.3	
Overweight/Obese (> 25)	15.9	14.9	

**Note:**

* Significant at p < 0.05.

### Factors associated with anemia

Table 3 illustrates the factors statistically significantly associated with anemia among women ageing between 13 and 40 years in Bangladesh. Variables that did not show significant correlation in the *χ*^2^ tests were removed from further analysis and the rest were entered in the binary regression model all at the same time. The results of regression analysis revealed a significant association with participant’s type of residency (Rural: p = 0.045; OR = 0.953, 95%CI = 0.840–1.082), wealth status, educational attainment (p < 0.001; OR = 1.276, 95%CI = 1.132–1.439), and level of food security (p < 0.001; 95%CI = 1.348–1.83). The effect of food insecurity remained significant even after controlling for the other variables associated with anemia, increasing the odds of being anemic by 57%. Women of poor (p < 0.001; 95%CI = 1.176–1.594) and average (p = 0.030; 95%CI = 1.017–1.398) wealth status were respectively 1.4 and 1.2 times more likely to have anemia compared to the highest wealth category.

**Table 3:** Factors associated with anemia among women ageing between 15 and 49 years in Bangladesh, 2011.

Variables	p-value	OR	95%CI
**Residence (Urban)**^a^			
**Rural**	**0.045**	0.953	0.840–1.082
**Wealth (Richest)**^a^			
Middle	0.030*	1.193	1.017–1.398
Poorest	< 0.001*	1.369	1.176–1.594
**Educational attainment (≥ 6 years)**^a^			
< 6 years	< 0.001*	1.276	1.132–1.439
**Food security level (secure)**^a^			
Insecure	< 0.001*	1.571	1.348–1.830
**Microcredit membership (No)**^a^			
Yes	0.090	0.900	0.798–1.016
**BMI (Normal)**^a^			
Overweight/Obese	0.131	0.887	0.760–1.036
Underweight	0.054	0.876	0.767–1.000

**Notes:**

a Reference category.

* Significant at p < 0.05.

## Discussion

Food insecurity and micronutrient deficiency especially anemia are major public health concern in Bangladesh (Akhtar, 2015). However, epidemiological evidences on the association between household food security and anemia is sporadic. Based on nationally representative data from the 2011 Demographic and Household Survey, this study demonstrates the association between food insecurity and anemia among women of childbearing age in Bangladesh. Among the participants, more than two-fifths were suffering from varying degrees of anemia and about one-fifth were living in food insecure conditions. Consistent with a Mexican study, our findings showed that women who reported food insecurity had a substantially higher likelihood of suffering from anemia compared to their food secure counterparts. Food insecurity can lead to anemia through inadequate intake of micronutrients (Skalicky et al., 2006), and through decreased concentration of the micronutrients which facilitate the bioavailability of iron (Fe) such as vitamin A and C, folate (Backstrand et al., 2002).

Inadequate intake of micronutrients in food insecure households can be a result of under-consumption of food, or overconsumption of energy-dense but nutrient-poor diet which are becoming increasingly cheaper sources of calorie for poor consumers (Drewnowski & Eichelsdoerfer, 2010; Drewnowski & Darmon, 2005). Gradual liberalization of the agri-food market along with the rapid expansion of local food industries have triggered a nutritional transition from home cooked food to more processed and away-from-home meals. In India, similar shifts in dietary pattern has been shown to be associated with lower intake of green leafy vegetables (GLV) and fruits, less iron rich diet, and higher prevalence of anemia (Balarajan, Fawzi & Subramanian, 2013). Though the consumption pattern of convenience food is likely to differ across age (higher among adolescents and adults) and region (higher among urban residents), the increasingly higher cost of healthy diet (fresh fruits and vegetables (FFV)) (Mayuree et al., 2013) may cause adverse health effects among poor households in rural areas as well.

As expected, wealth and educational status were also found to be significantly associated with anemia status among the participants. Educated individuals usually tend to be more concerned about personal health and exhibit better self-efficacy and adherence to healthy dietary and lifestyle habits (Frieden, 2010). Low socioeconomic and educational status have shown to be associated with poor adherence to healthy eating patterns and micronutrients deficiencies among women in Sub Saharan Africa (Bain et al., 2013). Due to their low educational and socioeconomic status, women in Bangladesh are particularly vulnerable to food insecurity and micronutrient deficiency and debilitating health impacts of both them and their children compared to males (Choudhury et al., 2000). Such disparities rooted to the social and familial values are hard to address and require broad-scale health education and awareness building projects. Nutrition programs targeting at maternal health could be integrated with nutrition education to encourage best practice among adult women and whole society. Previous studies have demonstrated a positive impact of nutrition education on increased intake of micronutrients (iron, absorbable iron, and vitamin C) among adolescent girls (Alaofè et al., 2009) and pregnant women. One Korean study reported that children of mothers with higher educational level were less likely to develop anemia and had increased consumption of iron rich diet (Choi et al., 2011). These finding suggest a greater policy emphasis on female education and socioeconomic empowerment programs.

Coupled with nutrition and dietary transition, Bangladesh is also experiencing an epidemiological transition where national disease burden is undergoing a decreasing share of communicable diseases with a rising prevalence of non-communicable disease, e.g. obesity/overweight, diabetes, and cardiovascular diseases (Bishwajit, 2015). Previous studies from different settings have demonstrated that anemia status and body weight have certain correlations (Qin et al., 2013), which implies that the changing trend in body weight and composition in a population may alter underlying causes of anemia, and may require modifications in intervention strategies. Our findings demonstrate that compared to women with normal weight status, overweight/obese women had lower odds of suffering from anemia. This finding is consistent with one conducted on adult women in China (Qin et al., 2013). While some studies found no significant relationship between anemia and BMI (Ugwuja et al., 2015), the association between underweight and anemia status remains less clear from previous researches. Future studies should attempt to address this lacking by using specific grades of BMI to explore the impact on different levels of anemia.

In conclusion, prevalence of anemia is high among Bangladeshi women. Our study found a positive association between household food insecurity and anemia among women of reproductive age. Evidence suggests that both anemia and food insecurity are serious challenges for population health and the overall development of the nation. In order to effectively address these issues, national food and nutrition policymaking should reinforce the supplementation and bio-fortification programs and consider subsidizing nutritious foods for poor consumers. Special emphasis should be given to keeping price of staple grains within an affordable range and encourage healthy eating pattern by making animal food and FFV more affordable. National anemia prevention programs should be integrated with those that targets women’s socioeconomic empowerment and promoting household food security.

Our study has several noteworthy limitations. Anemia level was not stratified due to negligible percentage of severe anemia. Participants who reported severe food insecurity were also too few to categorize separately. Data were collected from a survey which was conducted in 2011; hence, the prevalence of anemia and food insecurity might have changed. Due to the secondary nature of the dataset, we had no control over the selection and inclusion of covariates and their measurements.

## Abbreviations

BDHS: Bangladesh Demographic and Health Survey
BMI: Body Mass Index
HFIAS: Household Food Insecurity Access Scale
FFV: Fresh Fruits and Vegetables
HFI: Household Food Insecurity
WHO: World Health Organization.

## Additional Information and Declarations

### Data Deposition

The following information was supplied regarding data availability: Data is publicly available at http://dhsprogram.com/data/Using-Datasets-for-Analysis.cfm.
